# Cammidge's Pancreatic Reaction in the Urine

**Published:** 1908-05-23

**Authors:** 


### SPECIAL ARTICLE.

### CAMMIDGE'S PANCREATIC REACTION IN THE URINE.

We hold no brief for Cammidge's reaction; indeed we have not any very strong belief in it; but others have, and we have received so many inquiries from practitioners as to how the reaction is obtained that we take this opportunity of referring to it and of quoting the method from Cammidge's Arris and Gale Lecture of 1904. The reaction consists of two distinct parts, described as reaction A and reaction B respectively.

### Technique of Reaction A.

"The specimen of urine to be examined is filtered and ten cubic centimetres of the filtrate are poured into a small flask. One cubic centimetre of strong hydrochloric acid is added, and a funnel having been placed in the neck to act as a condenser the flask is placed on a sandbath and gently boiled for ten minutes after the first sign of ebullition is detected. A mixture of five cubic centimeters of the filtered urine and five cubic centimetres of distilled water is then poured into the flask, which is afterwards cooled in running water. The excess of acid is now neutralised by slowly adding four grammes of lead carbonate and, after standing for a few minutes to allow of the completion of the reaction, the urine is filtered through a moistened filter paper and the flask is washed out with five cubic centimeters of distilled water on to the filter. To the clear filtrate are now added two grammes of powdered sodium acetate and 0.75 gramme of phenylhydrazine hydrochlorate, and the mixture is boiled for from three to four minutes on the sand bath. The hot fluid is then poured into a test tube and allowed to cool undisturbed. After the lapse of a period, varying with the severity of the case from one to twenty-four hours, a more or less abundant flocculent yellow deposit is found at the bottom of the tube, and this, when examined under the microscope with a $\frac{1}{6}$th inch objective, is seen to consist of sheaves and rosettes of golden-yellow crystals. As the presence of sugar in the urine would obviously vitiate the results thus obtained, it is necessary before proceeding to the test to make sure that the untreated urine does not give a reaction with phenylhydrazine. This may roughly be done with Fehling's solution or some similar test, but it is better to carry out a control experiment with phenylhydrazine hydrochlorate and sodium acetate in the manner just described, omitting the preliminary boiling with hydrochloric acid. Should the control experiment reveal even a trace of sugar it must be removed by fermentation, with subsequent boiling to expel the alcohol formed, before the investigation is proceeded with.

"The presence of albumin in the urine is also liable to cause trouble, and it is best got rid of either by treatment with ammonium sulphate or by acidifying with acetic acid, heating, and filtering."

The results obtained by this method were found not to be absolutely trustworthy, although a useful aid in diagnosis, since a positive reaction was also obtained in patients suffering from certain other diseases where active tissue-change was taking place, such as cancer, adenitis, pneumonia, and so forth.

### Technique of Reaction B.

It is stated that the formation of crystals by reaction A is interfered with in inflammation of the pancreas by a preliminary treatment of the urine with perchloride of mercury, but that such treatment does not affect the appearance of the crystals in cases of cancer of the pancreas and the other conditions which give rise to a positive reaction A. Reaction B requires the following procedure:—

"Twenty cubic centimetres of the filtered urine are thoroughly mixed with half its bulk of a saturated solution of perchloride of mercury. After standing for a few minutes it is carefully filtered, and to ten cubic centimetres of the filtrate one cubic centimetre of strong hydrochloric acid is added. The mixture is then boiled for ten minutes on the sand bath, and subsequently diluted with five cubic centimetres of the filtrate from the mixed urine and perchloride of mercury solution and ten cubic centimetres of distilled water. After cooling it is neutralised with four grammes of lead carbonate, and the succeeding stages of the operation are carried out as in reaction A."

### The Rationale of the Reactions.

The relation between the pancreas and glycosuria is so well known (we do not mean to imply that the relations between glycosuria and the pancreas are equally well established) that whenever it is thought that the pancreas may be diseased one of the first things that the clinician looks for is the presence of sugar in the urine either occasionally or continuously. The idea underlying the above reactions seems to be that the urine, in pancreatic affections, need not contain actual sugar and yet may contain a precursor of the sugar—a precursor which ought not to be there in health. The principle of the reactions is to convert this precursor into sugar by boiling with hydrochloric acid, and then to test for the sugar so formed by the phenylhydrazine test in the ordinary way. The crystals obtained are not actually named in Cammidge's own description of his tests, but they seem to be those of phenylglucosazone as far as one can judge from the account of them.

### Comments on the Reactions.

Upon purely *a priori* grounds one would be inclined to think favourably of the idea and to believe that there was something in it. It would be too much to expect that the results would be what is called "pathognomonic"—that is to say, that they were entirely free from fallacies; but one might well look forward to getting assistance from the tests in conjunction with the rest of the clinical evidence in the case. As we have said, we do not in any way vouch for the value of the tests, however; we merely describe them as we have been asked to do by many of our readers. Each must form his own opinion for himself. We propose to give some favourable reports upon the tests, from St. Thomas's Hospital, at the end of these notes, and it is right to mention that Mayo Robson is said to place considerable reliance upon the presence or absence of the reactions as indications for or against operation in cases supposed to be pancreatic; but one's faith, it seems to us, becomes considerably shaken when one is asked to believe that the rates of solubility of the crystals obtained should also be determined before final conclusions are drawn.

### The Rates of Solubility of the Crystals.

The author of the reaction goes on to say that "careful observations of the crystals isolated from the urines of various types of pancreatic disease showed that, while there was a general resemblance, variations in form occurred. On classifying the crystals from a considerable number of cases it was found that those obtained in malignant disease were as a rule coarser and broader than those seen in simple inflammation, and that in the simple inflammation again the crystals from acute cases were on the whole somewhat finer than those seen in most instances of chronic inflammation. The lines dividing these types were not hard and fast, however, and although to the experienced eye a suggestive difference was discernible, great reliance could not be placed on a differential diagnosis based on the microscopical characters of the crystals alone. In the course of our further investigations a character of much greater value was discovered, and this was the differing rate of solubility of the crystals in dilute sulphuric acid. It was at first thought that this difference depended on the varying size of the crystals, but more extended experience proved that this was not the case; for although the coarser crystals took, as a rule, longer to dissolve completely than the finer ones, exceptions were met with and the time of solution appeared to be directly dependent on the intensity of the inflammatory changes in the gland. If the crystals obtained in reaction A be observed under the microscope whilst a 1 in 3 solution of strong sulphuric acid is being irrigated under the cover-glass, they will be seen to turn brown when the acid reaches them, and then to dissolve. In acute pancreatitis the interval that elapses between the first appearance of the brown colour and the complete solution of the crystals under observation varies from a few seconds to half or three-quarters of a minute; in chronic pancreatitis it extends from half to one and a half, or rarely to two, minutes; while the crystals of malignant disease as a rule do not completely disappear for from three to five minutes. To form a trustworthy opinion it is advisable to take the mean of several observations, so that slight variations unavoidably present in the conditions of the experiment may be neutralised and comparable results obtained. The crystals met with in non-pancreatic cases turn brown and dissolve in dilute sulphuric acid in a similar way to those obtained in pancreatic affections, and they most closely resemble those of chronic pancreatitis in their time of solution, which generally ranges round one minute. Phenylglucosazone crystals, with the strength of acid mentioned, only slowly assume the brown coloration, and take five minutes or more to dissolve.

"The time-limits mentioned are the result of the comparison of a large number of observations, and will as a rule be found to be correct. The exceptions are in most instances cases of malignant disease of the pancreas."

### Comments on the Above.

The rate of solubility of crystals, especially when the variations lie within comparatively narrow limits, must always be a very risky basis for deciding any clinical question on, especially so momentous a decision as that between performing and not performing laparotomy. Indeed, to the ordinary man it must seem that the rates of solubility discussed above afford no basis for any decision at all. It is clear that the crystals obtained by Cammidge's reaction must either have the same chemical constitution in different cases or else that their constitution varies according to the lesion present. This sounds a useless sentence, perhaps, but it is worth considering; for it can surely only be if the crystals are of different composition in different cases that their rates of solubility can really vary apart from accident; if, however, the crystals obtained from a case of chronic pancreatitis differ intrinsically from those in a case of acute pancreatitis, upon what can the intrinsic difference depend? A chronic pancreatitis may be the result of a preceding acute pancreatitis; if during the acute stage the crystals are one thing, and during the chronic stage the crystals are a different thing, at what point does the change take place? It seems much more probable that, granted that the reactions have truth in them, the crystals obtained are of the same composition whether the inflammation be acute, subacute, or chronic. If, however, the composition of the crystals is the same in different cases, their rates of solubility in sulphuric acid can depend upon physical conditions only, varying with the size of the crystals, the degree of concentration of the fluid they are in, the rate at which the sulphuric acid is irrigated under the cover-glass, the temperature of the slide, and so forth.

Cammidge's own dicta are as follows:—

I. If no crystals are found by either the "A" or the "B" method the pancreas is not at fault, and some other explanation of the symptoms must be sought.

II. If crystals are obtained by the "A" method, but not by the "B" reaction, active inflammation of the pancreas is present and surgical interference is generally indicated.

(*a*) The crystals obtained by the "A" method will in acute inflammation dissolve in 33 per cent. sulphuric acid in about half a minute.

(*b*) In chronic inflammation the crystals obtained by the "A" method will take one or two minutes to disappear.

III. If crystals are found in preparations made by both the "A" and the "B" methods there may be:—

(*a*) Malignant disease of the pancreas, when the crystals will as a rule take from three to five minutes to dissolve, and operation is inadvisable.

(*b*) A damaged pancreas due to past pancreatitis, when the crystals will dissolve in from one to two minutes.

(*c*) Some disease not connected with the pancreas, when the crystals dissolve in about one minute.

We cannot but think that Cammidge's reaction, if it is to be of assistance at all, must sink or float upon the question of obtaining, or not obtaining, crystals by it, quite apart from the rates of solubility of any crystals that may be obtained.

### The Clinical Results Obtained by the Use of Reactions A and B.

The reactions have been tried in many hospitals, and there are already numerous statistics upon the subject. We do not propose to give all these, but we feel that it is only just that we should report that many observers state that they have been greatly helped by the use of this additional laboratory test. At St. Thomas's Hospital Cassidy has collected together 25 cases, and his opinion seems to be that, taken in conjunction with all the rest of the clinical data that each case affords, Cammidge's reaction is distinctly useful; but he puts it in this way:—

I. The value of reaction B is doubtful; and no conclusions can be drawn from the degree of solubility of any crystals obtained by A or B.

II. If reaction A is positive in a case of suspected pancreatic disease, the probability of such disease being present is increased.

III. If reaction A is negative, it is unlikely that the pancreas is diseased; in no case in which a negative reaction was obtained was disease of the pancreas demonstrated.

### Summary.

Cammidge's reaction is probably too complicated to be carried out in private practice, so that it is likely that urines to be tested for it will mostly be sent to special laboratories. Cammidge's reaction as described by the author consists of three parts; namely, (1) Reaction A; (2) reaction B; (3) the rates of solubility of crystals obtained. There is evidence to show that of these three parts, only reaction A has any diagnostic value; so that if reaction A is all that one need trouble about, the test is really not a very complicated one, and there are many who hold the opinion that reaction A, according as it is positive or negative, affords evidence of the presence or absence of pancreatic disease.

